# Evaluation of a neural network‐based photon beam profile deconvolution method

**DOI:** 10.1002/acm2.12865

**Published:** 2020-03-30

**Authors:** Karl Mund, Jian Wu, Chihray Liu, Guanghua Yan

**Affiliations:** ^1^ Department of Radiation Oncology University of Florida Gainesville FL USA

**Keywords:** artificial neural network, deconvolution, detector response function, volume averaging effect

## Abstract

**Purpose:**

The authors have previously shown the feasibility of using an artificial neural network (ANN) to eliminate the volume average effect (VAE) of scanning ionization chambers (ICs). The purpose of this work was to evaluate the method when applied to beams of different energies (6 and 10 MV) and modalities [flattened (FF) vs unflattened (FFF)], measured with ICs of various sizes.

**Methods:**

The three‐layer ANN extracted data from transverse photon beam profiles using a sliding window, and output deconvolved value corresponding to the location at the center of the window. Beam profiles of seven fields ranging from 2 × 2 to 10 × 10 cm^2^ at four depths (1.5, 5, 10 and 20 cm) were measured with three ICs (CC04, CC13, and FC65‐P) and an EDGE diode detector for 6 MV FF and FFF. Similar data for the 10 MV FF beam was also collected with CC13 and EDGE. The EDGE‐measured profiles were used as reference data to train and test the ANNs. Separate ANNs were trained by using the data of each beam energy and modality. Combined ANNs were also trained by combining data of different beam energies and/or modalities. The ANN's performance was quantified and compared by evaluating the penumbra width difference (PWD) between the deconvolved and reference profiles.

**Results:**

Excellent agreement between the deconvolved and reference profiles was achieved with both separate and combined ANNs for all studied ICs, beam energies, beam modalities, and geometries. After deconvolution, the average PWD decreased from 1–3 mm to under 0.15 mm with separate ANNs and to under 0.20 mm with combined ANN.

**Conclusions:**

The ANN‐based deconvolution method can be effectively applied to beams of different energies and modalities measured with ICs of various sizes. Separate ANNs yielded marginally better results than combined ANNs. An IC‐specific, combined ANN can provide clinically acceptable results as long as the training data includes data of each beam energy and modality.

## 1 INTRODUCTION

In the commissioning of a treatment planning system (TPS) and the periodic quality assurance (QA) of a linear accelerator (linac), it is essential to accurately measure the transverse photon beam profiles produced by the linac.^1^ It is well‐known that the measurements, typically performed with a finite‐size ionization chamber (IC), are compromised by the volume averaging effect (VAE).^2^ The VAE, caused by the signal averaging over the detector's active volume, can artificially broaden the penumbra of photon beam profiles by 2–3 mm, depending on the detector's effective size. This effect has profound implications for the planning, delivery, and QA of radiotherapy using small beam segments, such as intensity‐modulated radiotherapy (IMRT) and volumetric‐modulated arc therapy (VMAT).^3^, ^4^, ^5^, ^6^, ^7^ For example, Yan et al demonstrated that the elimination of the VAE enabled the use of stricter criteria (from 3%/3 mm to 2%/2 mm) in patient‐specific IMRT QA, which in turn led to higher chances of detecting dosimetric errors arising from either treatment planning or delivery system.^4^, ^7^, ^8^

Direct reconstruction of the “true” beam profiles from the measurements is the preferred approach to address the VAE.^9^ A process called deconvolution is performed where the VAE, modeled with a detector response function, is numerically or analytically removed from the measurement based on the convolution theorem.^9^, ^10^, ^11^ Photon beam profile deconvolution is challenging for a few reasons. First, since the penumbra of photon beam profiles is located in the high gradient area, Fourier‐based numerical deconvolution methods suffer from high‐frequency measurement noise. Second, each type of IC has a unique detector response function that is hard to determine. Additionally, there is no consensus on the exact shape and extend of the detector response function.^12^, ^13^ Third, the shape of the beam profiles varies with beam geometry (depth and field size) and beam modality [flattening‐filter (FF) vs flattening‐filter‐free (FFF)], which makes it difficult to fit the beam profiles with functions that can facilitate analytical deconvolution.^4^, ^14^, ^15^

In a previous proof‐of‐principle paper, the authors investigated the feasibility of photon beam profile deconvolution using an artificial neural network (ANN).^10^ The ANN used a sliding window to extract inputs from the measurement and output of the deconvolved value at the center of the window. It was demonstrated that the ANN achieved great performance for 6 MV photon beam profiles measured with a CC13 IC.

The aim of this study was twofold. First, we evaluated the performance of the ANN‐based deconvolution method when applied to photon beam profiles of different beam energies, of different beam modalities, and measured with ICs of different sizes. The effectiveness of the method under different scenarios needs to be extensively evaluated before it can be introduced into clinical use. Secondly, we determined whether an IC‐specific combined ANN was sufficient for clinical use. To that end, we compared the performance of ANNs separately trained for each beam energy/modality with that of ANNs trained by combining data of different energies and/or modalities.

## 2 MATERIALS AND METHODS

### 2.A Neural network model

**Fig. 1:**
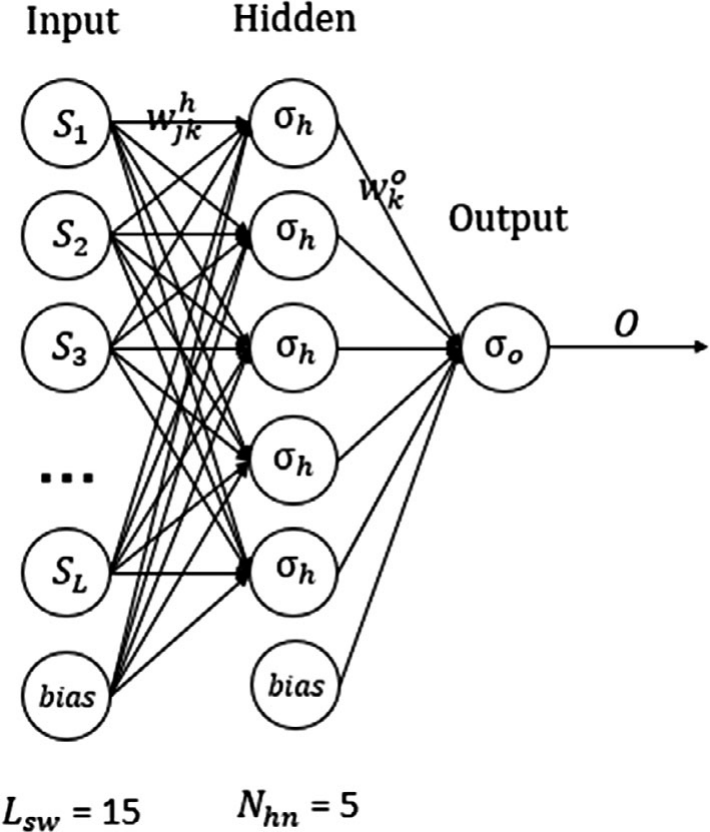
The three‐layer artificial neural network consists of an input layer with 15 nodes, a hidden layer with five nodes, and an output layer with a single node. The input signals *S_i_* (i = 1, 2, …, 15) are extracted from the measured beam profile with a sliding window. It outputs one deconvolved value at a time, corresponding to the center of the sliding window.

Here, we briefly describe the ANN model, the detail of which can be found in our previous paper.^10^ The ANN consists of an input layer, a hidden layer, and an output layer (Fig. 1). While the input and hidden layers have multiple nodes, the output layer has a single node. A sliding window is used to extract inputs from the measured beam profile at 1 mm resolution. The output, corresponding to the deconvolved value at the center of the window, is given by

$$O=\sigma_{o}\left(\sum_{k=1}^{N_{\mathrm{hn}}}w_{k}^{o}\sigma_{h}\left(\sum_{j=1}^{L_{\mathrm{sw}}}w_{\mathrm{jk}}^{h}s_{j}+b_{k}^{h}\right)+b^{o}\right),$$

where s denotes input signal from the measured profile; w represents the weight associated with the link connecting adjacent layers; b represents the bias of the hidden or output neurons. The deconvolved beam profile is created in a point‐by‐point fashion with the sliding window moving across the measured beam profile. The hidden nodes and output node use the hyperbolic tangent sigmoid activation function ($\sigma_{h}$) and linear activation function ($\sigma_{o}$), respectively. The number of input nodes ($L_{\mathrm{sw}}$, i.e., the size of the sliding window) and the number of hidden nodes ($N_{\mathrm{hn}}$) were determined with a parameter sweeping algorithm in our previous paper. It was found that $L_{\mathrm{sw}}=15$ and $N_{\mathrm{hn}}=5$ yielded the best performance and these parameters were used in this study.

### 2.B Data collection

Transverse beam profiles produced by an Elekta linac (Versa HD, Elekta Inc., Crawley, UK) were measured with a three‐dimensional cylindrical water tank (Sun Nuclear Corp. Melbourne, FL). The measurement geometries included seven field sizes (2 × 2, 3 × 3, 4 × 4, 5 × 5, 6 × 6, 8 × 8, 10 × 10 cm^2^) at four depths (1.5, 5, 10, and 20 cm), totaling 28 beam profiles. We limited our study to small fields as the penumbra change caused by VAE is on the order of 1–2 mm, which is more significant for small fields than for large fields (e.g., 20 × 20 cm^2^). These measurements were performed for the 6 MV FF and FFF beams with three ICs (CC04, CC13, FC65‐P, IBA Dosimetry, Schwarzenbruck, Germany) and an EDGE diode detector (Sun Nuclear Corp. Melbourne, FL). The FFF beam was measured with a dose rate of 600 MU/min, instead of the default high dose rate (1400 MU/min). The beam profiles of the 10 MV FF beam were also measured with the CC13 and the EDGE. All the measurements were smoothed and resampled to 1 mm resolution. The CC04 has a radius of 2 mm and an effective volume of 0.04 cm^3^. While the radii of the CC13 and FC65‐P are both 3 mm, the effective volume of FC65‐P is five times larger than that of CC13 (0.65 vs 0.13 cm^3^). Compared to the ICs, the EDGE diode has significantly less VAE due to its small effective measuring area (0.8 × 0.8 mm^2^).^4^, ^9^

### 2.C Network training

There are practical advantages of training and using as less ANNs as possible. Therefore, it is of great interest to know whether a separate ANN is needed for each scenario (beam energy, beam modality, and IC). Theoretically, the “real” beam profile can be expressed as the convolution between the measured beam profile and a deconvolution kernel, and the ANN is trained to simulate the convolution operation. The deconvolution kernel is directly related to the detector response function. ICs of different types have different detector response functions due to their differences in radius, effective volume, and physical construction.^16^ Therefore, separate ANNs are warranted for different types of ICs. The remaining questions are, for the same IC, do we train one ANN for two beam energies combined or separate ANN for each beam energy? Do we train one ANN for two‐beam modalities combined or separate ANN for each beam modality? For an IC, is it feasible to train just one ANN that works for all energies and modalities?

To answer these questions, we compared the detector response function of an IC under different beam energies and different beam modalities. The measured beam profile $P_{m}\left(x\right)$ is the result of convolving the “true” beam profile $P_{t}\left(x\right)$ with the detector response function $K_{\sigma }\left(x\right)$, which is typically approximated using a Gaussian function with a shape parameter σ.^7^, ^11^ Given a pair of $P_{m}\left(x\right)$ and $P_{t}\left(x\right)$, σ can be determined through iterative optimization,

$$\sigma =argmin_{\sigma }{\left(P_{m}\left(x\right)-P_{t}\left(x\right)⊗K_{\sigma }\left(x\right)\right)}^{2}$$

where ⊗ denotes the convolution operation. Practically, for $P_{m}\left(x\right)$ and $P_{t}\left(x\right)$, we use the same beam profile measured with the IC and the EDGE, respectively.^11^, ^17^, ^18^ The EDGE‐measured beam profile can be regarded as the “true” beam profile due to its negligible VAE.^19^, ^20^, ^21^ Here, we determined σ for CC13, the most commonly used scanning IC, for the 6 MV FF beam, the 6 MV FFF beam, and the 10 MV FF beam. For each beam energy/modality combination, we calculated σ for each geometry (field size and depth) separately, resulting in 28 shape parameters.

Our study revealed that there were subtle differences in the shape parameters for different beam energies and modalities. The impact of the differences on the ANN performance could not be understood unless separate and combined ANNs were compared directly. Therefore, we trained separate ANNs as references, then trained combined ANNs for comparison. A separate ANN was trained for each beam energy, modality, and measuring IC combination. The 28 beam profiles were divided into non‐overlapping training, validation, and test datasets. The training and validation datasets included 12 profiles for the 2 × 2, 4 × 4, 6 × 6, and 10 × 10 cm^2^ fields at 1.5, 10, and 20 cm depths; the test dataset consisted of the remaining 16 beam profiles (the beam profiles at 5 cm depth for all seven fields and the ones at the other three depths for 3 × 3, 5 × 5, and 8 × 8 cm^2^). The training and validation datasets were used to train and optimize the weights and biases of the ANN, which minimized the mean square error (MSE)

$$MSE=\frac{1}{N}\sum_{i=1}^{N}{\left(O_{i}-P_{i}\right)}^{2}$$

between the predicted output $O_{i}$ and the desired value $P_{i}$ taken from the EDGE‐measured profile (N is the length of the profile). The standard Levenberg–Marquardt backpropagation algorithm was used in the training. The network was initialized with random weights and biases and the training was repeated 10 times with each attempt involving 400 epochs. The network with the smallest MSE was selected for evaluation. The test dataset, unseen by the ANN, was used to test its generalization ability. In addition to MSE, another metric called PWD (penumbra width difference) was used in the evaluation. The PWD was calculated as the difference in the penumbra width (distance between 20% and 80% intensity)

$$PWD=\left|W_{o}-W_{r}\right|$$

where $W_{o}$ and $W_{r}$ were the penumbra width of the deconvolved and the reference profile, respectively. While the MSE evaluates the overall agreement between two profiles, the PWD focuses on the penumbra area where the VAE is most prominent. Note that, in this work, the EDGE‐measured profiles are used as references in PWD calculation by virtue of the negligible VAE associated with the EDGE.

Three combined ANNs were also trained for comparison. We firstly trained an ANN for two‐beam modalities (6 MV FF and 6 MV FFF) combined. The training, validation, and test datasets were formed by combining the profiles from the 6 MV FF beams and the 6 MV FFF beams. Similarly, an ANN was trained for two beam energies (6 and 10 MV) combined. Finally, an ANN was trained by combining all the CC13‐measured profiles from the 6 MV FF, 6 MV FFF, and 10 MV FF beams. The performance of the combined ANNs was compared with that of the separate ANNs.

## 3 RESULTS

**Fig. 2:**
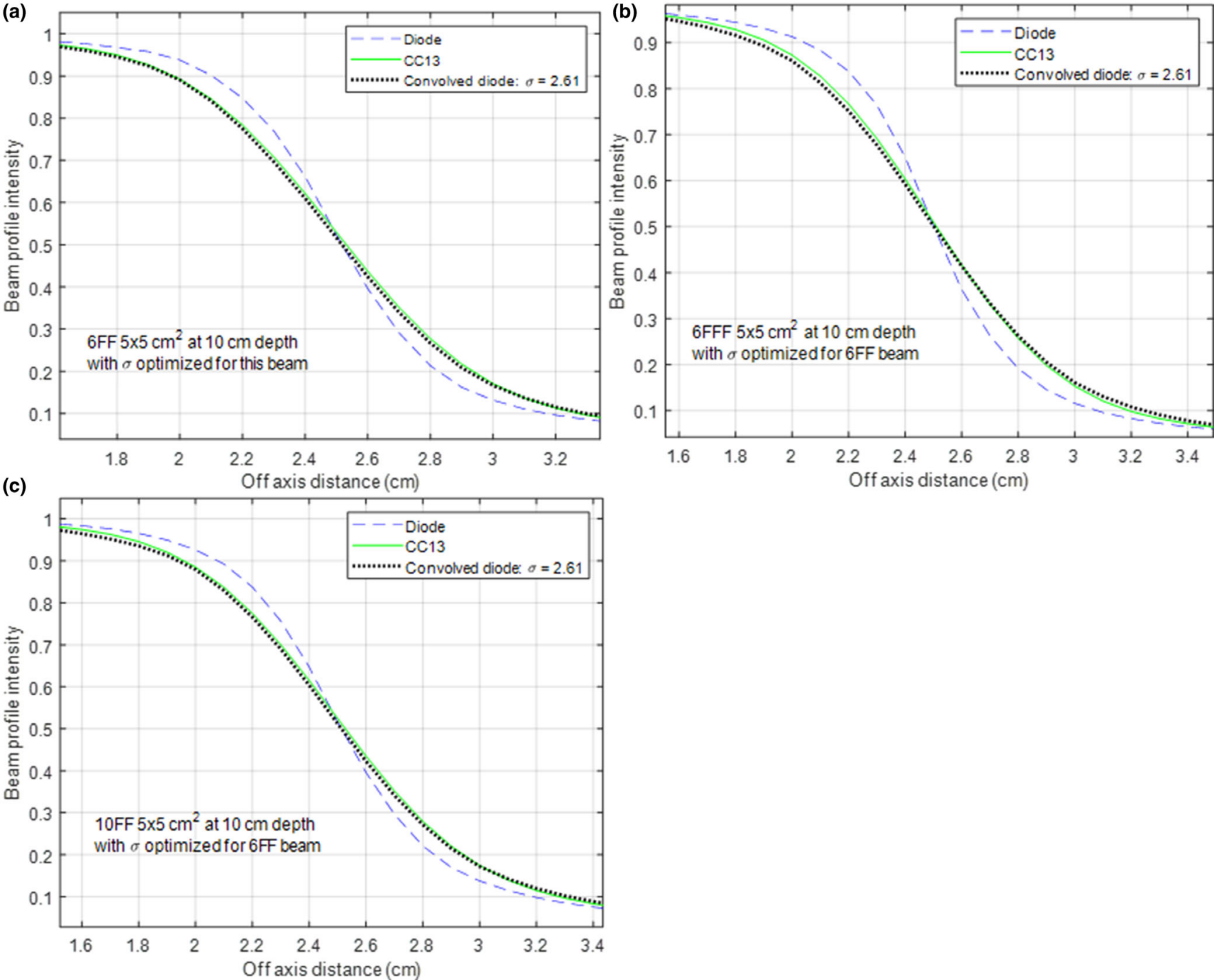
Influence of beam energy and modality on the detector response function of CC13. Using the 6 MV FF beam (5 × 5 cm^2^ and 10 cm depth), the shape parameter of the detector response function was determined to be 2.61 mm. The diode‐measured, CC13‐measured and convolved profiles are shown for 6 MV FF (a), 6 MV FFF (b), and 10 MV FF (c).

The average shape parameter of the CC13's detector response function for 6 MV FF, 6 MV FFF, and 10 MV FF beam was 2.56 ± 0.06, 2.93 ± 0.10, and 2.70 ± 0.07 mm, respectively. Figure 2 illustrates the difference with the beam profile of a 5 × 5 cm^2^ field at 10 cm depth. In Fig. 2(a), the shape parameter of the detector response function for the 6 MV FF beam was determined to be 2.61 mm. The detector response function was then used to convolve the diode‐measured profile of the 6 MV FFF and 10 MV FF beam. While the convolved result agreed with the CC13 measurement pretty well for the 10 MV FF beam [Fig. 2(c)], the difference was noticeable for the 6 MV FFF beam [Fig. 2(b)]. The difference between the penumbra width of the convolved profile and that of the CC13 measured‐profile was 0.04 mm for the 10 MV FF beam and 0.41 mm for the 6 MV FFF beam.

**Fig. 3:**
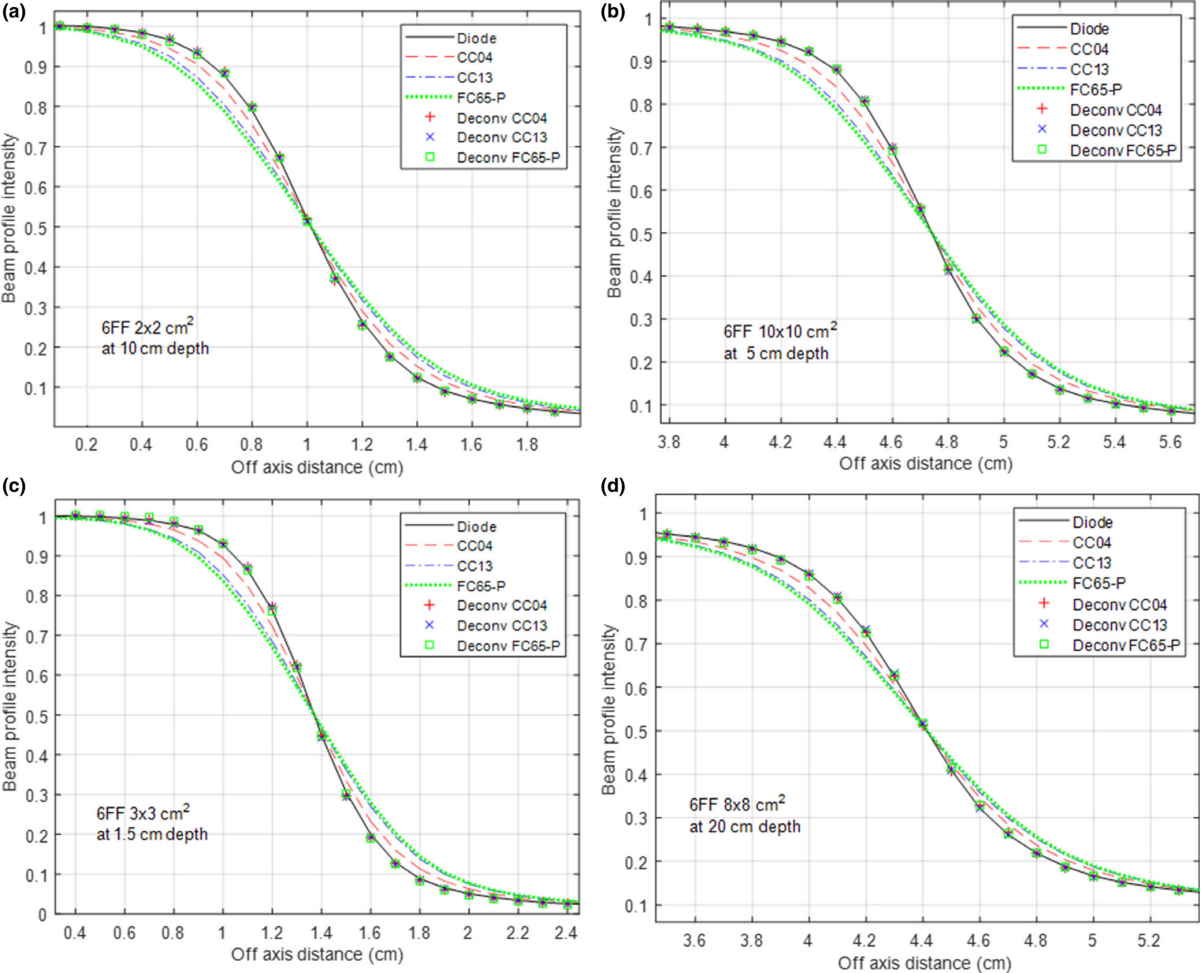
Performance of the artificial neural network for a 6 FF beam, separately trained for each of the three ionization chambers (CC04, CC13, and FC65‐P). The diode‐measured beam profiles were used as references. The results on training data are shown in (a); the results on test data are in (b–d).

Figures 3 shows the performance of the three ANNs separately trained for CC04, CC13, and FC65‐P for the 6 MV FF beam with a few examples. Figures 3(a)–3(d) shows the results for training data and test data, respectively. Excellent agreement between the deconvolved and the diode‐measured profile was achieved for all three ICs at all studied geometries. After deconvolution, the mean PWD for CC04, CC13, and FC65‐P was reduced from 0.98 ± 0.13, 1.90 ± 0.11, and 2.23 ± 0.13 mm to 0.09 ± 0.07, 0.05 ± 0.04, and 0.07 ± 0.06 mm, respectively.

**Fig. 4:**
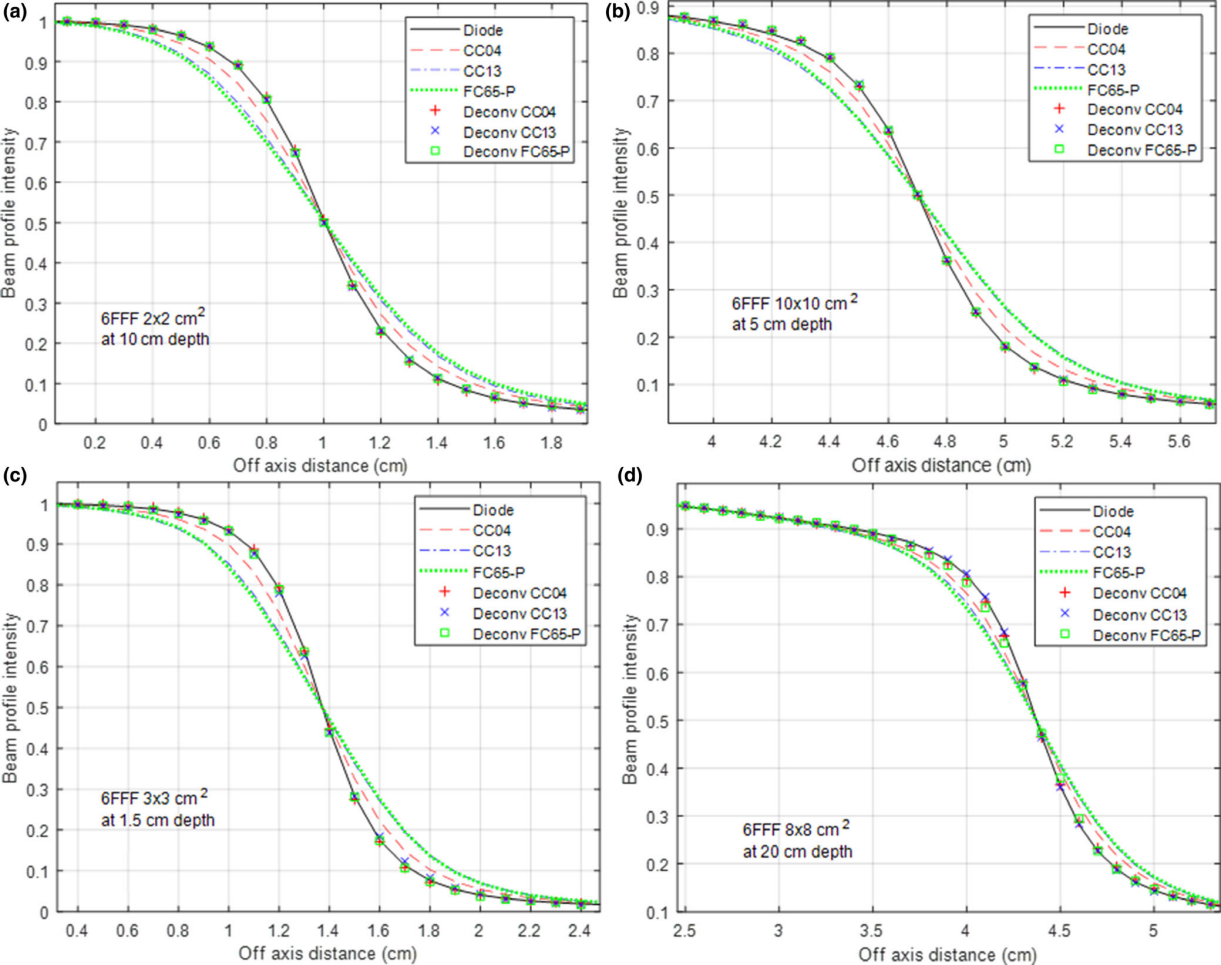
Similar to Figure 3, but with the 6 FFF beam. The results on training data are shown in (a); the results on test data are in (b–d).

Figure 4 shows similar results, except for the 6 MV FFF beam. Generally, good agreement between the deconvolved and the diode‐measured profiles was observed for all three ICs. The ANN for FC65‐P showed slightly larger deviations at large field sizes (8 × 8 cm^2^). After deconvolution, the mean PWD was reduced from 1.31 ± 0.23, 2.46 ± 0.23, and 2.55 ± 0.20 mm to 0.15 ± 0.09, 0.10 ± 0. 08, and 0.13 ± 0.12 mm, respectively, for the CC04, CC13, and FC65‐P.

Table 1 details the PWD before and after deconvolution for a few selected beam geometries (both 6 MV FF and 6 MV FFF) with examples for all three ICs. In general, the ANN trained for the 6 MV FF beam had slightly better performance than that for the 6 MV FFF beam. After deconvolution, the maximum PWD was 0.32 mm for the 6 MV FF beam (8 × 8 cm^2^ at 1.5 cm depth with CC04) and 0.52 mm for the 6 MV FFF beam (8 × 8 cm^2^ at 20 cm depth with FC65‐P, shown in Fig. 4(d)).

**Table 1:** Penumbra width difference (PWD) of the ionization chamber‐measured beam profiles and the artificial neural network‐deconvolved beam profiles with respect to the diode‐measured profiles for 6 MV FF and FFF beams.

**Depth (cm)**	**PWD OF 6 MV FF BEAMS**						**PWD OF 6 MV FFF BEAMS**					
	Training data		Testing data				Training Data		Testing data			
	6 × 6 cm^2^		3 × 3 cm^2^		8x8 cm^2^		6x6 cm^2^		3x3 cm^2^		8x8 cm^2^	
	**CC04**	**Deconv**	**CC04**	**Deconv**	**CC04**	**Deconv**	**CC04**	**Deconv**	**CC04**	**Deconv**	**CC04**	**Deconv**
**1.5**	1.00	0.08	0.85	0.08	1.23	0.32	1.37	0.13	1.10	0.04	1.60	0.30
**5**	1.09	0.10	0.88	0.04	1.19	0.21	1.43	0.06	0.96	0.19	1.63	0.26
**10**	0.98	0.00	0.87	0.05	1.08	0.09	1.46	0.04	1.04	0.24	1.69	0.25
**20**	1.21	0.19	0.90	0.07	1.10	0.00	1.58	0.13	1.00	0.27	1.78	0.38
	**CC13**	**Deconv**	**CC13**	**Deconv**	**CC13**	**Deconv**	**CC13**	**Deconv**	**CC13**	**Deconv**	**CC13**	**Deconv**
**1.5**	2.00	0.09	1.87	0.00	2.04	0.10	2.46	0.08	2.37	0.18	2.74	0.23
**5**	2.07	0.16	1.88	0.00	2.08	0.09	2.47	0.06	2.26	0.11	2.77	0.09
**10**	1.94	0.04	1.81	0.01	2.03	0.04	2.40	0.13	2.38	0.15	2.67	0.06
**20**	1.96	0.02	1.75	0.04	1.96	0.01	2.73	0.08	2.39	0.15	2.7	0.08
	**FC65‐P**	**Deconv**	**FC65‐P**	**Deconv**	**FC65‐P**	**Deconv**	**FC65‐P**	**Deconv**	**FC65‐P**	**Deconv**	**FC65‐P**	**Deconv**
**1.5**	2.23	0.00	2.20	0.05	2.53	0.29	2.66	0.09	2.50	0.03	2.98	0.36
**5**	2.34	0.04	2.19	0.04	2.40	0.14	2.66	0.05	2.33	0.13	2.85	0.21
**10**	2.26	0.08	2.11	0.03	2.35	0.07	2.62	0.04	2.38	0.16	2.85	0.36
**20**	2.31	0.11	1.98	0.08	2.23	0.04	2.67	0.25	2.25	0.14	2.92	0.52

**Fig. 5:**
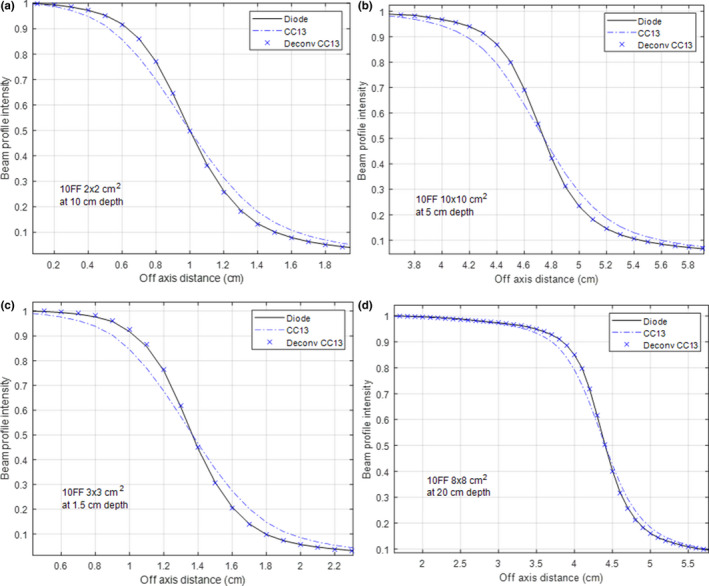
Performance of the artificial neural network for the 10 MV FF beam measured with CC13. The training data is in (a); the test data in (b‐d). The diode‐measured beam profiles are used as references.

Figure 5 shows the results of the ANN for the 10 MV FF beam measured with CC13. Fig. 5(a) shows the results for the training data and Figs. 5(b)–5(d) shows the results for the test data. Excellent agreement was also achieved for all the studied beam geometries. The mean PWD decreased from 1.99 ± 0.15 mm to 0.07 ± 0.05 mm after deconvolution. The reduction of PWD is detailed in Table 2. After deconvolution, the PWD was under 0.20 mm for all studied beam geometries.

**Table 2:** Penumbra width difference (PWD) of the CC13‐measured beam profiles and the artificial neural network‐deconvolved beam profiles with respect to the diode‐measured profiles for the 10 MV FF beam.

Depth (cm)	PWD OF 10 MV FF BEAMS					
	Training data					
	6 × 6 cm^2^		3 × 3 cm^2^		8 × 8 cm^2^	
	CC04	Deconv	CC04	Deconv	CC04	Deconv
1.5	1.82	0.09	2.09	0.05	2.24	0.10
5	1.73	0.19	2.17	0.15	2.18	0.09
10	1.72	0.05	2.03	0.02	2.11	0.01
20	1.69	0.04	1.94	0.04	2.12	0.07

Depth (cm)	Testing data					
	CC13	Deconv	CC13	Deconv	CC13	Deconv
1.5	1.90	0.11	2.11	0.14	2.18	0.04
5	1.94	0.06	1.98	0.01	2.14	0.04
10	1.91	0.03	1.93	0.00	1.96	0.15
20	1.81	0.01	1.98	0.00	2.08	0.06

**Fig. 6:**
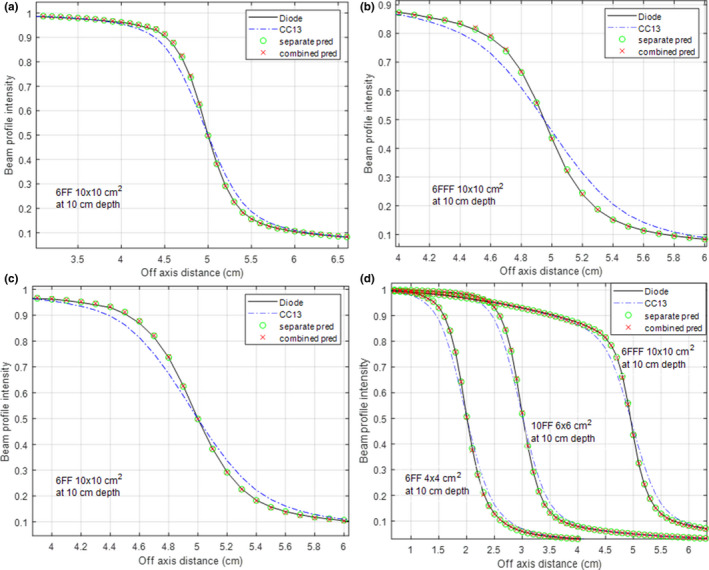
Comparison between separate artificial neural networks (ANNs) and combined ANNs. The combined ANN was trained by combining data from 6 MV FF and 6 MV FFF in (a) and (b), combining data from 6 MV FF and 10 MV FF in (c), and combining data from 6 MV FF, 6 MV FFF and 10 MV FF in (d).

Table 3 compares the performance of the combined ANNs with that of the separate ANNs. Listed in the table are the mean PWDs of all the 28 beam profiles either measured with IC or deconvolved with separate or combined ANNs. Wherever data is not available (the 10 MV FF data were only measured with CC13 and EDGE), it is flagged with “n/a.” The results indicated that the separate ANNs had slightly better performance than the combined ANNs. With separate ANNs, the mean PWD of deconvolved profiles was within 0.15 mm for all beam energies, modalities, and ICs; with combined ANNs, the mean PWD was under 0.20 mm, except the CC13‐measured 6 MV FFF data, where the ANN trained for combined modalities had a mean PWD of 0.24 mm. Figure 6 illustrates the difference using CC13‐measured data. In Figs. 6(a)–6(b), the combined ANN was trained for two modalities combined (6 MV FF and 6 MV FFF). Compared with separate ANNs, the combined ANN slightly overestimated or underestimated the penumbra. However, the clinical impact should be insignificant given that the magnitude of difference is negligible. In Fig. 6(c), the combined ANN was trained for two energies combined (6 MV FF and 10 MV FF). In this example, the separate ANN and the combined ANN had nearly identical performance. In Fig. 6(d), the combined ANN was trained with data combined from 6 MV FF, 6 MV FFF, and 10 MV FF beams measured with CC13. It achieved satisfactory results for both beam energies and modalities.

**Table 3:** Comparison between separate and combined artificial neural networks (ANNs). Shown in the table are the penumbra width difference (PWD) before and after deconvolution. Separate ANNs are trained for each beam energy, modality, and ionization chamber combination. Combined ANNs are trained for two modalities combined (6FF + 6FFF), two energies combined (6FF + 10FF), or two modalities and energies combined (6FF + 6FFF + 10FF). “n/a” indicates that the data is not available (10 MV FF beam was only measured with CC13).

Energy/modality	IC	PWD — IC	PWD — separate ANN	PWD — combined ANN		
				6 FF + 6 FFF	6 FF + 10 FF	6 FF + 6 FFF + 10 FF
6 MV FF	CC04	0.98 ± 0.13	0.09 ± 0.07	0.11 ± 0.07	n/a	n/a
	CC13	1.90 ± 0.11	0.05 ± 0.04	0.10 ± 0.07	0.06 ± 0.04	0.11 ± 0.06
	FC65‐P	2.23 ± 0.13	0.07 ± 0.06	0.11 ± 0.06	n/a	n/a
6 MV FFF	CC04	1.31 ± 0.23	0.15 ± 0.09	0.19 ± 0.12	n/a	n/a
	CC13	2.46 ± 0.23	0.10 ± 0.08	0.24 ± 0.14	n/a	0.19 ± 0.12
	FC65‐P	2.55 ± 0.20	0.13 ± 0.12	0.16 ± 0.12	n/a	n/a
10 MV FF	CC13	1.99 ± 0.15	0.07 ± 0.05	n/a	0.08 ± 0.06	0.12 ± 0.07

## 4 DISCUSSIONS

There has been a recent surge in applying machine learning techniques to address challenges in medical physics.^22^ Powerful and innovative neural network models have been proposed to assist tumor detection,^23^ image segmentation,^24^, ^25^ treatment planning,^26^, ^27^ and treatment outcome prediction^28^ with success. In our work, we successfully trained simple ANNs to mitigate the VAE associated with ICs that are commonly used for transverse beam profile scanning. We evaluated the performance of the method when applied to beams of different energies or modalities and measured with ICs of various sizes. Satisfactory results were achieved for each studied beam energy, modality, and IC with both separate and combined ANNs. Artificial neural networks excel at a variety of tasks, thanks to the representation power associated with the hidden neurons in the hidden layers, which enables well‐trained ANNs to closely approximate any continuous functions. We have shown that, in the deconvolution problem, the desired outcome (“true” beam profile) is essentially a function of the measured beam profile.^10^ Therefore, we can expect that ANN is suitable for the deconvolution problem even the function involves a complex, not well‐defined deconvolution kernel.

The successful training of an ANN relies on the abundancy of training data, especially for fully connected, large‐scale ANN.^22^ In this work, the three‐layer ANN has 15 input nodes and 5 hidden nodes, totaling 86 parameters (80 weightings and 6 biases). We used only 12 beam profiles to train the ANN and found that it generalized well on the unseen test data. The success could be attributed to the relatively simple architecture of the ANN and the similarity in the data. For IC‐measured data (input to the ANN) and diode‐measured data (desired output of the ANN), the difference only lies in the penumbra area as characterized using PWD. For the 6 MV FF beam data measured with the same IC, the mean PWDs were within 0.3 mm across all the studied field sizes (ref Table 1); for the 6 MV FFF and 10 MV FF data, they were within 0.6 and 0.4 mm of each other, respectively. For the same field size, the PWD varied <0.3 mm from 1.5 to 20 cm depth. This similarity was also reflected in the shape parameters σ of the detector response function. Between different energies and modalities, the mean σ varied <0.4 mm. For the same energy and modality, the standard deviation of σ across all studied geometries was within 0.1 mm. This came as no surprise for two reasons. First, it is well known that IC has nearly no energy dependence.^1^ Second, as far as the impact of beam modality on IC response is concerned, it has been reported that ICs may be subject to excessive ion recombination in the high dose rate mode (e.g., 1400 MU/min) of FFF beams.^29^ However, since our data for the FFF beams were collected in the normal dose rate mode (600 MU/min), the beam modality had no influence on the detector response. Note that we were not concerned with the penumbra of beam profiles, which varied as beam energy, modality, and geometry (field size and depth) changed. Instead, we were interested in the difference in the penumbra of the same beam profile, measured with different detectors (IC vs diode). The difference was mainly attributed to the response difference of the IC, dominated by the VAE. The high‐level similarity in the training, validation and test data contributed to the success of the ANNs, which were trained to mitigate the difference.

We can also use the variation of the detector response function’s shape parameter to explain the comparison between separate and combined ANNs. Separate ANN was trained for specific beam energy and modality, where the shape parameters had very little variations; combined ANN was trained for mixed beam energies or modalities, where the shape parameters had slightly larger variations. Therefore, while separate ANN had excellent performance, combined ANN performed slightly worse. However, since the overall variation in σ was limited, the performance of combined ANNs was still acceptable. The clinical implication is that, for a particular IC, it is sufficient to train just one combined ANN, which works for all beam energies and modalities. It is worth pointing out that the training data must include data from each energy and modality. This is because ANNs can interpolate but cannot extrapolate. When the input is outside the scope defined by the training data, the output of the ANN is not reliable.

Note that the combined model was trained with combined data from different beam energies and modalities, but no information regarding the energy and modality was used as input to the ANN. In this sense, the combined model acted more like an averaged model, which treated beam profiles from different energies and modalities in the same way. Adding energy or modality information to the ANN input may improve its performance. The model could learn to handle different beam setup distinctively through the learning process with the involvement of data from different setups. However, since the performance of the combined ANN was deemed satisfactory, the improvement may be marginal. Therefore, such attempt was not made in this study.

To our knowledge, this is the first report studying the mitigation of VAE in FFF photon beam profiles. The unique shape of unflattened beam profiles presents challenges to analytical deconvolution methods. These methods use analytical functions such as the difference of error functions to fit the beam profiles,^4^, ^15^, ^30^ which is feasible for flattened beam profiles but challenging for unflattened beam profiles. However, it does not pose a problem for our ANN‐based method. It learns how to mitigate the difference between IC‐measured data and diode‐measured data, which occurs mainly in the penumbra area even for unflattened beams. Both separate and combined ANNs achieved excellent results for FFF beams. Not only was the penumbra area restored, the unflattened shape of the beam profiles was also well preserved.

## 5 CONCLUSIONS

We evaluated the robustness of an ANN‐based photon beam deconvolution method when applied to photon beams of different energies, of different modalities, and measured with ICs of various sizes. For each IC, excellent results were achieved with ANNs separately trained for each energy and modality combination. Clinically acceptable results were also achieved with ANNs trained by combining both beam energies and modalities. Therefore, for a given IC, an IC‐specific, combined ANN is sufficient for clinical use as long as the training data includes beam profiles from each energy and modality.

## CONFLICT OF INTEREST

The authors have no conflicts to disclose.
